# Homœopathy; an Examination of Its Doctrines and Evidences

**Published:** 1852-02

**Authors:** 


*Homœopathy: An Examination of its Doctrines and Evidences.* By WORTHINGTON HOOKER, M. D. pp. 146. New York, Charles Schribner, 1851.

Those of our readers, who are familiar with the British journals of medicine, must have doubtless been struck with the attention which has lately been bestowed in this quarter on *homœopathy.* The apostacy of one or two individuals, occupying prominent positions in the British medical world, has probably been the immediate stimulus of the interest thus displayed. But the fact is manifest, that our brethren in the British islands are disposed now to deal with the charlatanism in question, as an antagonist which must be seriously and efficiently met. We confess that we have viewed this movement with much satisfaction. It has long been our impression, that the contemptuous indifference with which the progress of the humbug has been generally treated by the profession, particularly with us, has been bad policy. That it has taken a hold upon a portion of the educated and refined classes of society, cannot be denied. And absurd as we deem its doctrines and pretensions, they have rallied partisans whom it is worth while to convince, or at least expose. It is, therefore, with especial cordiality, that we welcome Dr. Hooker's admirable little brochure on this subject.

Dr. Hooker is so well known to the profession as one of our ablest and most judicious politico-ethical writers, that any thing from his pen must at once commend itself to general interest and attention. The essay before us is "the Fiske Fund Prize Dissertation of the Rhode Island Medical Society," on the subject, "Homœopathy, so called, its history, and refutation." And we may say of it, most unreservedly, that it places our profession under the strongest obligations to the accomplished author. In style, force, and temper, it is truly excellent, and must, we feel, do much good.

Dr. Hooker's examination of homœopathy, crushing as it is in analysis and argument, is, we think, particularly likely to prove useful, from its great fairness and moderation of tone. The author professes to have "no desire to search out its weak points and leave untouched its strong ones, if there be any, but to be willing to meet it at every point." And this is certainly the right way to obtain a hearing among the intelligent and candid, who have adopted the system of Hahnemann, "without a real understanding of its merits." Those who will give Dr. Hooker a hearing, it may be safely asserted, "will be induced to abandon the combination of falsities and inconsistences which this system presents."

In concluding this notice, we ask the attention of our readers to the following remarks in relation to medical fallacies in general:

> "Medical delusions, generally, though so diversified in their forms, have a strong family resemblance, and the fallacies of Homœopathy may be considered as the types of other fallacies. An exposition of them, therefore, will reveal to the reader the foundations of other delusions and forms of quackery, and will perhaps enable him so to apply the principles of evidence in medicine, that he may in future the more readily detect error, whether it appear in the garb of learning or of ignorance.
>
> A refutation merely of Homœopathy, without regard to other delusions, or to the general sources of error, would be a comparatively trivial, and almost useless effort. If it should be successful in dislodging this boasted system from its hold upon the popular belief and favor, some other fallacious system would take its place. And if left to itself, it would in a little time pass away, like all other delusions before it. In attacking Homœopathy therefore, we must look beyond this delusion, and aim at an exposure of the common sources of error, if we wish to produce any valuable and permanent effect."

